# UV light-based reprocessing of flexible endoscopes without working channel in Oto-Rhino-Laryngology: an effective method?

**DOI:** 10.1007/s00405-021-06737-1

**Published:** 2021-03-13

**Authors:** Stefan A. Rudhart, Frank Günther, Laura Dapper, Kruthika Thangavelu, Urban W. Geisthoff, Petar Stankovic, Thomas Wilhelm, Boris A. Stuck, Stephan Hoch

**Affiliations:** 1 Department of Otolaryngology, Head and Neck Surgery, University Hospital Marburg, Philipps-Universität Marburg, Baldingerstrasse, 35043 Marburg, Germany; 2 Department of Medical Microbiology and Hygiene, University Hospital Marburg, Philipps-Universität Marburg, Marburg, Germany; 3 Department of Otolaryngology, Head/Neck and Facial Plastic Surgery, Sana Kliniken Leipziger Land, Borna, Germany; 4 Medical Faculty, Philipps-Universität Marburg, Marburg, Germany

**Keywords:** Hygiene, Sterilization, Disinfection, Microbiology

## Abstract

**Background:**

Reprocessing of flexible endoscopes (FEs) is often expensive, time consuming, and becomes increasingly complex, due to rising demands of hygiene. After beneficial results in reprocessing of rigid endoscopes using Impelux™ UV-C light technology, we tested the same method for reprocessing of FEs without working channel.

**Materials and methods:**

Testing was performed on FEs without working channel after routine clinical use (transnasal flexible endoscopy). Disinfection consisted of mechanical precleaning and 60 s exposure to Impelux™ UV-C light technology. Bacterial contamination was tested on 50 FEs before and after disinfection. Further 50 FEs regarding protein residuals. The absolute effectiveness of the D60 was tested on 50 test bodies (RAMS) with a standardized contamination of 10^7^ colony-forming units (CFU) of Enterococcus faecium.

**Results:**

The FEs were contaminated with a high average value of 916.7 CFU (± 1057 CFU) after clinical usage. After reprocessing, an average contamination of 2.8 CFU (± 1.6) on 14% (*n* = 7) of the FEs was detected consisting of non-pathogenic species, the remaining FE were sterile. After reprocessing, all FEs were protein-free (< 1 μg). The artificially contaminated test bodies showed no remaining bacterial contamination after disinfection, resulting in an average absolute germ reduction of about 10^7^ CFU.

**Conclusion:**

Impelux™ UV-C light technology efficiently reduces bacterial contamination of FEs and might be useful in daily practice.

## Introduction

Flexible endoscopes (FEs) without working channel are an integral part of all endoscopic examinations in various medical disciplines. Within the field of otorhinolaryngology (ORL), they are often used for the clinical examination of the upper aero-digestive tract in terms of transnasal flexible endoscopy. In this context, FEs are especially suitable for patients which are difficult to examine due to an enhanced pharyngeal reflex. In the clinical routine of an ORL-outpatient department, FEs are often used and reprocessed multiple times a day, which is time consuming and associated with a risk of loss or damage of the endoscopes during processing and transportation. According to the results of a previous study on rigid endoscopes, extensive contamination, including pathogenic bacteria, can be assumed after clinical use of the endoscopes in ORL [1]. Hence, the reprocessing of FEs is highly important to prevent transmission of pathogens between the patients. Therefore, the prevention of transmission events is mostly related to an insufficient reprocessing between the patients examinations [2]. In the last years, several outbreaks with highly resistant pathogens revealed the relevance of a reliable reprocessing of the endoscopes [3]. According to the Spaulding`s classification, system of medical equipment at least a high-level disinfection is required for endoscopes without working channel due to their classification as semi-critical patient care devices [4–7]. Hence, a large number of high-level disinfection methods are applied for reprocessing of endoscopes in ORL and, up to now, no standard has been implemented. However, reprocessing of FEs on high-level standards gets increasingly complex due to the increasing number of multi-resistant bacteria and the resulting demands of hygiene. Up to now, established methods for reprocessing of FEs are often costly and time consuming and sometimes lacking standardizability. Therefore, new, cheaper and faster methods are required for daily use in an ORL with a high volume of patients, without compromising the safety and quality of reprocessing.

Previous studies have revealed satisfactory results regarding surface disinfection by UV light. In this context, its effectiveness against problematic hospital-acquired-germs or biofilm-building bacteria has to be highlighted [8, 9]. Surface disinfection by UV light is known for more than 120 years. Nils Ryberg Finsen, a Danish physician, was one of the first who successfully treated bacterial infections in patients by UV light. He even was awarded with the Noble Prize for Medicine in 1903 for successfully treating tuberculosis of the skin by UV light [10, 11]. Several years later, in the 1930′s, UV lamps became commercially available and were widely used in medicine after the Second World War. After 1945, disinfection agents were not commonly available in the medical sector, therefore, UV lights were used in permanent operation for prevention of bacterial outbreaks in medical facilities [12]. Due to continuous improvements in technology, UV light has now a wide application range, e.g. it is used worldwide for disinfection of natural drinking water, where it is preferred, as it does not influence its natural taste or smell characteristics [13]. We have previously tested a UV light system for the reprocessing of rigid endoscopes with promising results. In this study, an absolute bacterial reduction on standardized test bodies of about 10^6^ CFU was observed. Furthermore, in clinical practice, nearly all endoscopes were sterile and practically protein-free after reprocessing [1]. To our knowledge, UV light systems have not been analyzed for the reprocessing of FEs in ORL to date. In contrast to the D25, the D60 is specially developed for bigger sized endoscopes as FEs in ORL. Thus, in this trial, we analyzed the efficiency of UV-C light in the disinfection of FEs without working channel using the D60 UV light system.

## Materials and methods

### Reprocessing of endoscopes

The present study was performed at a tertiary care unit. We included the whole spectrum of patients with different ORL diseases, including infectious and non-infectious patients to analyze a representative cross-section of used endoscopes. The tested UV light system was analyzed in an everyday clinical use scenario. In this study, we tested the D60 UV light system (UV Smart, Delft, Netherlands) for reprocessing of non-channel FEs 2.5 × 270 mm, (KARL STORZ SE and Co. KG, Tuttlingen, Germany) with a plastic surface and a steerable tip used within the field of ORL.

The examined FEs were reprocessed by mechanically precleaning for 20 s with a water-soaked tissue. To create these precleaning wipes, we used a box with 100 polyester dry wipes (Schülke Wipes Safe and Easy, Schülke GmbH, Norderstedt, Germany) and filled the box with 2 L of distilled and sterilized water (Ampuwa, Fresenius Kabi Germany GmbH, Bad Homburg, Germany). After precleaning, the air-dried endoscopes were hung in the D60 and got exposed to UV light for further 60 s. Each endoscope was disinfected separately after usage. After UV light exposure, the residual contamination on the endoscopes was evaluated. Finally, in order not to compromise patient safety, each endoscope was reprocessed with a washer-disinfector (WD425E, Belimed, Zug, Switzerland) in accordance with a standardized protocol.

### Microbiological examination/protein testing of endoscopes

First, 50 FEs were tested on trypticase soy agar-based surface contact samples (Merck Millipore, Darmstadt, Germany) directly after clinical use (posterior rhinoscopy/laryngoscopy) to evaluate the bacterial contamination on the endoscopes. Then, 50 further FEs were tested by contact sampling after endoscopic examination and reprocessing by water-based precleaning and UV light exposure in the D60. Microbiological tests were taken from the first 12.5 cm of the shaft, due to the length of the contact samples, adjusting the tip of the endoscope a neutral 0 degrees position. The tip and shaft of the examined endoscope were fixed on the ager plate by a sterile tweezer to avoid partial lifting of the endoscope from the test plate. The test plates were incubated at 37 °C for 1 week. Afterwards, matrix-assisted laser desorption ionization time-of-flight mass spectrometry (Bruker Daltonik, Bremen, Germany) was used for microbiological identification. Third, protein residues were tested on 50 endoscopes after clinical use and reprocessing by precleaning and UV exposure using the Medi-Check™ (Hygiena Medisafe GmbH, Wentorf, Germany) test-kit. These tests for protein contamination were used as tracer for prion- and viral load on the endoscopes after usage on the patient. Results were reported by change of color, according to the detected amount of protein contamination on the endoscope. After incubation at 55 °C for 15 min, the range of the test-kit represented a contamination from 0 (light green) to 50 μg (dark grey/black).

### Testing of bactericidal reduction on standardized RAMS test bodies

The effectiveness of disinfection by the D60 was evaluated under standardized conditions with 10 cm stainless-steel test bodies, contaminated with bacterial load of approximately 8 × 10^7^ CFU of *Enterococcus faecium*. 52 test bodies were used: 50 were reprocessed with the D60 and 2 were used to prove the initial bacterial contamination on the test bodies (control samples). The so-called “RAMS” contamination on the surface of the test bodies consists of *Enterococcus faecium*, corn starch, bovine albumin and mucein to simulate an organic contamination on the endoscope. RAMS test bodies are commonly used in testing of reprocessing devices for instruments of the highest category in Spaulding’s classification system [5]. Hence, the testing conditions using RAMS test bodies are equal with the highest reprocessing standards in medicine. Methodically, the test body was hung inside the D60 with a metal wire, in the same position as the endoscope is usually placed for reprocessing. The metal wire had no contact with the microbiologically examined parts of the test body and furthermore, the test body did not touch the surface of the D60. The RAMS test bodies were reprocessed in the same way as the tested endoscopes (water-based precleaning and UV-C exposure by the D60). To avoid interference by shadowing, each test body was cleaned separately.

### The D60 UV light system

**Fig. 1:**
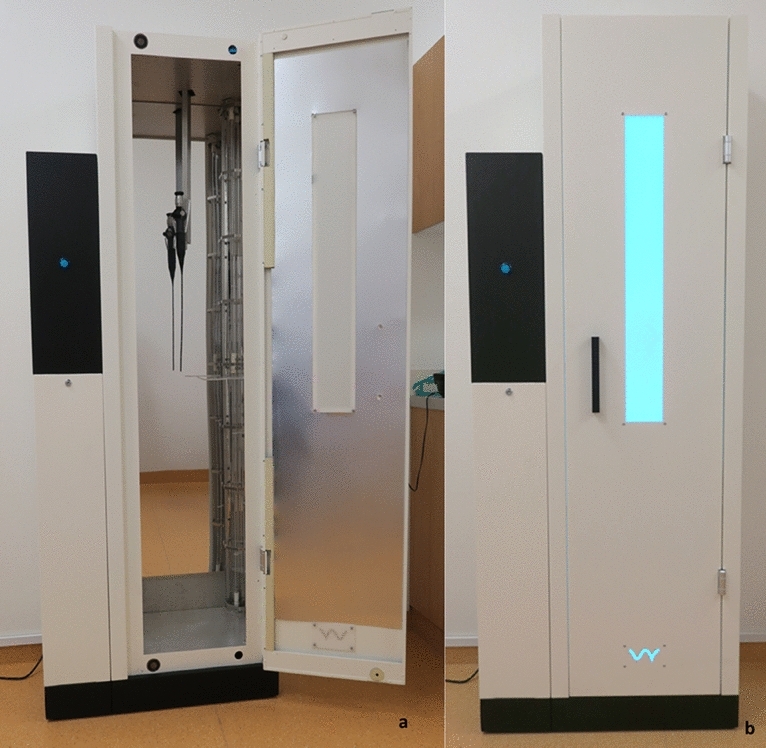
**a** Arrangement of a FE in the D60. **b** Sealed D60 while reprocessing a FE

**Fig. 2:**
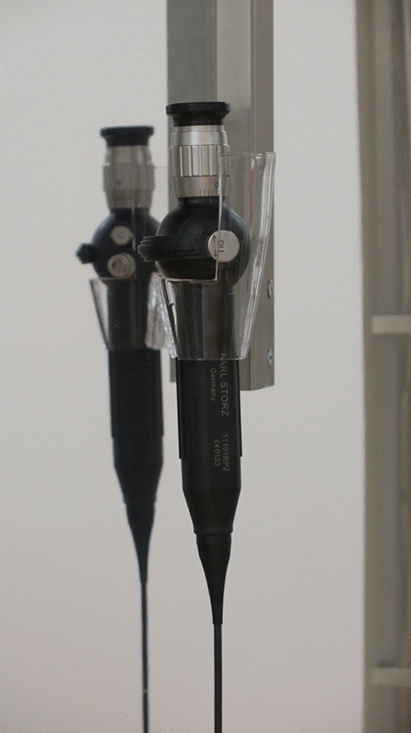
Close-up of the endoscope holder with a FE

The UV Smart D60 UV light system uses UV-C light for the disinfection of medical endoscopes within 1 min. Except the water-based precleaning, no further detergents or liquids are needed for reprocessing endoscopes by the D60. The endoscopes should not exceed a maximum length of 120 cm to fit into the disinfection chamber (Fig. 1a). The holder for the endoscopes in the D60 is made of transparent glass which allows the UV light to reach the endoscope surface (Fig. 2). For prevention of the carcinogenic UV radiation, the device is sealed while reprocessing. Hence, while applying UV light, the radiation is not able to escape the D60 (Fig. 1b). In accordance to UV Smart’s trials, the D60 light system reaches a log-5 bacterial reduction within 1 min. Therefore, the D60 was preset for a disinfection time of 1 min.

The UV technology used in the D60 light system (UV Smart Impelux™ technology) works at a wavelength of 253.7 nm. An application dose of 1962 J/m^2^ is delivered in each cycle. According to internal investigations of the manufacturer, the applied UV-C dose does not affect the surface of the endoscope. Nevertheless, it causes DNA and RNA destruction in irradiated microorganisms on the endoscope. Dirt, debris and grime are not penetrable for the germicidal effect of the UV-C light. Hence, all endoscopes must be optically clean before using the UV light system.

### Statistics and ethical approval

Excel 2019 (Microsoft Corporation, Redmond, Washington, USA) was used for statistical descriptive analysis. According to the statement of the ethics committee, a formal approval was not needed, as neither patients nor personal data were included. However, this study was notified to the ethics committee of the Medical Faculty of the Philipps-Universität Marburg.

## Results

**Fig. 3:**
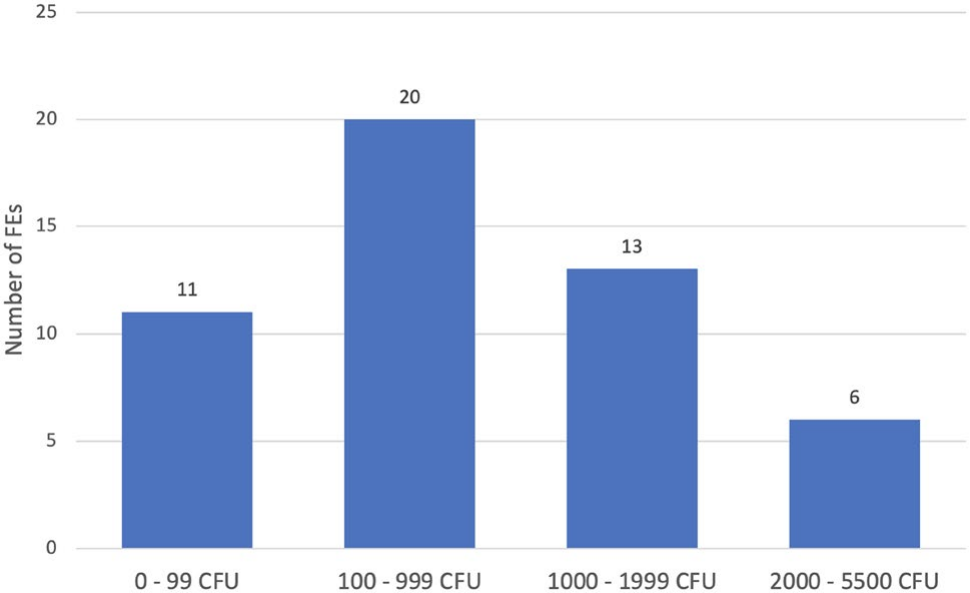
Number of FEs depending on the contamination on their surface after clinical usage in the patient without disinfection

Directly after clinical use without any kind of reprocessing a high contamination was found on the FEs. The mean value on all 50 endoscopes was 916.7 CFU (± 1057; 10–5500 CFU) (Fig. 3). A highly variable bacterial flora was found on the FEs, including germs of the permanent mucosal flora (e.g. *Coagulase Negative Staphylococci*) as well as potentially pathogenic bacteria (e.g. *Klebsiella spp.*). The bacterial cultures identified on the microbiological samples and the frequency of detection are shown in Table 1.

**Table 1:** Identified bacteria and number on the FEs after clinical use before UV exposure

Identified bacteria	Number (%)
Coagulase negative *Staphylococcus*	45 (90%)
*Micrococcus luteus*	16 (32%)
*Staphylococcus aureus*	13 (26%)
*Neisseria species*	8 (16%)
*Klebsiella aerogenes*	7 (14%)
*Bacillus species*	6 (12%)
*Corynebacterium species*	5 (10%)
*Viridans streptococci*	2 (4%)
*Klebsiella oxytoca*	1 (2%)

The FEs reprocessed by the D60 showed an average contamination of 0.28 CFU. A bacterial contamination was found on seven (14%) FEs (1 CFU in six cases and 8 CFU in one case; ø 2 CFU). The bacteria found on the FEs after reprocessing can all be attributed to the mucosal microflora (*Coagulase Negative Staphylococci*, *Micrococcus luteus*, *Bacillus spp.* and *Corynebacterium spp.*). The remaining 43 (86%) endoscopes were sterile after reprocessing (0 CFU).

After water-based precleaning and UV-C disinfection, all of the 50 tested FEs were nearly protein-free (< 1 μg). Only 2 FEs showed a minimal contamination (< 1 μg), while the other 48 FEs were protein-free (0 μg).

No further bacterial contamination (0 CFU) was found on the 50 standardized RAMS test bodies after reprocessing. The two control samples still were contaminated with 8 × 10^7^ CFU, which results in a germ reduction of about 10^7^ for the reprocessed test bodies.

## Discussion

In the context of cross-contamination caused by used endoscopes and several healthcare-associated infections with multi-resistant pathogens, new and especially safe ways of surface disinfection might by highly relevant in the future [3]. As shown in a recent study regarding the D25 UV light system, the Impelux™ technology for UV-C disinfection could be a suitable method for handling these problems [1]. However, a device reprocessing FEs by UV light has not been investigated to date. Therefore, we tested the D60 UV light system for reprocessing of FEs, showing results similar to the study on the D25 UV light system and fulfilling the requirements for a successful reprocessing between different patients. However, the results for the D60 (ø 0.28 CFU) were nearly as good as the results for the D25 (ø 0.12 CFU). It is noticeable that only one endoscope was contaminated with more than 1 CFU, which may be explained by an artificial contamination of this FE after reprocessing. In this context, the present study was performed in an outpatient department under clinical conditions. Therefore, handling with the FEs under unsterile conditions after reprocessing may further have influenced the results. Another explanation for the slightly higher contamination found in this study might be the differences in the surface texture between rigid and flexible endoscopes. In contrast to FEs, the surface of rigid endoscopes is made of stainless steel and consists of fewer components. Thus, design-specific characteristics of the FEs such as microscopic gaps between different components might prevent sufficient precleaning and disinfection by UV light. Furthermore, FEs are more difficult to preclean, due to their surface properties and missing mechanical resistance.

Generally, UV radiation destroys the cell’s DNA and RNA, by forming thymine dimers. As a result, gene expression and replication is prevented and the irradiated cells die by apoptosis [14]. It is even efficient against otherwise problematic- and multi-resistant pathogens [6, 7, 12], Gram-positive and -negative bacteria, fungal and bacterial spores [15]. This can be explained by the fact that resistance mechanisms or biofilm-building abilities have no influence on the effectiveness of UV light. However, it is generally known that UV light also has a carcinogenic effect in irradiated organisms due to its physical mode of action on the DNA and RNA. Especially, skin cancer due to UV radiation by the sun is a commonly problem for humans [16]. Hence, the operator requires a sufficient shielding when the device is applying UV light for disinfection reasons. In the device examined here, the shielding against UV radiation is achieved by a box-shaped design as well as a sealing of the device while applying UV light. However, due to its physical properties, UV light does not penetrate solids or not transparent liquids. In the context of ORL, these substances or fluids are represented by secretions of the upper airways, solid mucus or blood. Therefore, it is important to remove this gross contamination by a type of precleaning from the FE before using a UV-based disinfection method. Otherwise, reprocessing of the FE could be insufficient. However, due to its influence on the overall result, the importance of precleaning must be given special weight when examining a new reprocessing method. Therefore, a water-based wipe without any microcide, disinfectant or enzymatic components was used, in order not to interfere with the results by any precleaning agents. Furthermore, we chose a precleaning method that is also cost-effective, practicable and nearly everywhere available.

To date, there are no data in the literature regarding the expectable contamination on FEs after clinical usage within the field of ORL. Only one study analyzed the bacterial contamination on rigid 30° and 70° ORL endoscopes. A high bacterial load, with an average value of 66,908 CFU on the endoscopes was found in this study [1]. In the present study, we also found a high bacterial contamination (ø 916.7 CFU) on the tested FEs after clinical use with a high variety of contamination between the different FEs. However, several factors caused by the patient himself and the examination might influence the amount of contamination on the endoscopes. In this context, mainly the extent of mucosa-contact, the bacterial colonization on the mucosa and the varying infectivity between the patients have to be considered. The germs found correspond mostly to the typical mucosal flora of the upper airways [17]. Nevertheless, it has to be considered, that besides bacteria of the normal mucosal flora also facultative pathogenic bacteria have been found on the FEs after clinical use without disinfection. These pathogenic bacteria are able to cause severe, even life-threatening complications in some patients [4, 18].

For the standardized RAMS test bodies, an absolute bacterial log reduction factor (LRF) of 7 (from 8 × 10^7^ CFU to 0 CFU) was found in the present study. By reaching an average reduction of LRF 7, the D60 fulfilled the requirements for semi-critical devices defined by the Commission on Hospital Hygiene and Infection Protection at the Robert Koch Institute (RKI) and the Federal Institute for Drugs and Medical Devices (BfArM) in Germany [19]. In the present study, carrier substances as bovine albumin, mucein and corn starch were used which are normally used for testing of reprocessing methods for critical devices. Hence, the results suggest the potential of the tested device in reprocessing of even more difficult contaminations. The results for the standardized test body tests did not fluctuate during the test series. Therefore, it seems that the tested UV system delivers consistent reprocessing quality. In addition, the electronically controlled disinfection process ensures standardized and documented reprocessing cycles.

In addition, the quality of precleaning might have influenced the results. While several studies have investigated the importance of precleaning in UV-based disinfection methods, this issue remains controversial. Nevertheless, there is a tendency in the literature, that precleaning has a rather small effect on the final reprocessing result [20–23]. Furthermore, due to the physical properties of UV radiation, it must be assumed that protein residues are mainly reduced by the mechanical precleaning, but not by the UV light itself.

Some studies found the distance of the UV lamp to the object and further shadowing as one key factor for its performance [15, 24]. In the D60, this distance is relatively short and the light distribution seems to be efficient and prevents shadowing. Nevertheless, it cannot be ruled out that shadowing could have occurred in microscopically small gaps between the components of the FE. In contrast, the RAMS test bodies had a completely flat stainless-steel surface which prevents shadowing effects.

In case of endoscope reprocessing, besides efficiency, economic aspects are of importance. The time required for reprocessing and the associated personnel costs must be taken into account. In comparison to other reprocessing methods for FEs, a reprocessing cycle with the D60 is associated with minimal costs. In addition, the possibility for point-of-care disinfection offers additional advantages. The D60 works with a reprocessing cycle of about 2 min including precleaning, drying and disinfection.

## Conclusion

Reprocessing of endoscopes is a complex and time consuming as well as cost-intensive process. In this context, the D60 UV light system showed good disinfection results in a routine clinical setting. In light of its potential for fast and simple point-of-care disinfection, it offers substantial advantages to standard disinfection methods for FE without a working channel.

## Data Availability

Available, if requested.
